# Antidiarrheal Activity of* Dissotis multiflora* (Sm) Triana (Melastomataceae) Leaf Extract in Wistar Rats and Subacute Toxicity Evaluation

**DOI:** 10.1155/2017/4038371

**Published:** 2017-11-05

**Authors:** Alian Désiré Afagnigni, Maximilienne Ascension Nyegue, Chantal Florentine Ndoye Foe, Youchahou Njankouo Ndam, Frédéric Nico Njayou, Marie Christine Fonkoua, François-Xavier Etoa

**Affiliations:** ^1^Laboratory of Microbiology, Department of Microbiology, Faculty of Science, University of Yaoundé I, Yaoundé, Cameroon; ^2^Laboratory of Phytobiochemistry and Medicinal Plants Study, Department of Biochemistry, Faculty of Science, University of Yaoundé I, Yaoundé, Cameroon; ^3^Laboratory of Pharmacology and Toxicology, Department of Biochemistry, Faculty of Science, University of Yaoundé I, Yaoundé, Cameroon; ^4^Laboratory of Bacteriology, Centre Pasteur of Cameroon, Yaoundé, Cameroon

## Abstract

The present work was undertaken to evaluate antidiarrheal activity of ethanolic leaf extract of* Dissotis multiflora* (Sm) Triana* (D. multiflora) *on* Shigella flexneri*-induced diarrhea in Wistar rats and its subacute toxicity. Diarrhea was induced by oral administration of 1.2 × 10^9^ cells/mL* S. flexneri *to rats. Antidiarrheal activity was investigated in rats with the doses of 111.42 mg/kg, 222.84 mg/kg, and 445.68 mg/kg. The level of biochemical parameters was assessed and organs histology examined by 14 days' subacute toxicity.* S. flexneri *stool load decreased significantly in dose-dependent manner. The level of ALT increased (*p* < 0.05) in male rats treated with the dose of 445.68 mg/kg while creatinine level increased in rats treated with both doses. In female rats, a significant decrease (*p* < 0.05) of the level of AST and creatinine was noted in rats treated with the dose of 222.84 mg/kg of* D. multiflora*. Histological exams of kidney and liver of treated rats showed architectural modifications at the dose of 445.68 mg/kg. This finding suggests that* D. multiflora* leaf extract is efficient against diarrhea caused by* S. flexneri* but the treatment with doses lower than 222.84 mg/kg is recommended while further study is required to define the exact efficient nontoxic dose.

## 1. Background

Diarrhea is an alteration of a normal bowel movement characterized by an increase in the water content, volume, or frequency of stools [1]. According to a world estimation of the 2015 world records, 5.9 million deaths occurred in children less than five years of age. About half of these deaths were caused by infectious diseases and conditions such as pneumonia, diarrhea, and measles [2]. Due to unhygienic livelihood conditions, peoples of the third world countries are very prone to several common diseases including diarrhea [3]. The major causative agents of diarrhea in humans include* Campylobacter* sp.,* Salmonella* sp.,* Escherichia coli, Shigella* sp.,* Yersinia* sp., and* Candida albicans* [4, 5].* S. flexneri *is the most common species that actually presents a great public health burden because it is endemic and is the major causative agent of dysentery in developing countries, and WHO has estimated that it causes 10% of acute diarrhea in children less than 5 years old [4].

Nowadays, most of the current antibiotics have considerable limitations in terms of antimicrobial spectrum and side effects and their wide spread overuse has led to increasing clinical resistance of previously sensitive microorganisms and to the occurrence of uncommon infections [6]. In order to overcome the menace of diarrhea in developing countries, especially the discomfort and inconvenience of frequent bowel movements, the WHO has introduced a program for diarrheal control which involves the use of traditional herbal medicines [7]. Several African medicinal plants have been reported to be useful in the treatment, management, and/or control of diarrhea by traditional healers [8]. However, the safety and therapeutic potentials of some of these medicinal plants have not been validated yet [9]. Among them,* Dissotis multiflora* (Sm) Triana* (D. multiflora)* is one of the popular medicinal plants being used in the traditional medicine.

*D. multiflora* is a plant belonging to the family of Melastomataceae. The genus* Dissotis* comprises about 140 species in Africa [10]. They are climbing shrubs, shrubs, or small trees of up to 2 m and are found in some African countries such as Democratic Republic of Congo, Benin, Nigeria, Ivory Coast, and Cameroon [11].* D. multiflora* leaves are used in Cameroon folk medicine to treat diarrhea without evidence of their antidiarrheal activity and toxicological status. Previous work has reported the* in vitro* antibacterial and antioxidant activities of ethanolic extract of* D. multiflora* and reported that this plant contains alkaloids, flavonoids, tannins, steroids, and others most of which do have known antidiarrheal activity [12, 13]. Indeed, no previous studies have revealed its antidiarrheal and toxicological properties. However many plants of the* Dissotis* genus were screened for antibacterial and antidiarrheal activities and toxicity [14–19]. Proof of antidiarrheal and toxicological assessment of* D. multiflora* leaf extract is necessary. Therefore, the present study was undertaken to evaluate the antidiarrheal activity of ethanolic extract of* D. multiflora* on* Shigella flexneri*-induced diarrhea in rat models and its subacute toxicity.

## 2. Material and Methods

### 2.1. Collection and Identification of Plant Materials

*D. multiflora* plant was collected at Nkoupa-Matapit in the Western region of Cameroon in December 2013. The plant identification was done at the Cameroon National Herbarium by comparison with specimen number 20950/HNC of* Dissotis multiflora* Triana (Melastomataceae).

### 2.2. Extraction Procedure

The leaves of* D. multiflora* were air-dried for one week at room temperature and weighed. The samples were then ground to a fine powder in a mortar and 200 g of dried powder of each sample was soaked for 48 hours in 600 mL of ethanol 95%. The mixing was filtered with Whatman number 1 filter paper and concentrated using a Rotavapor (Buchi) at 55°C (yield: 13,32%). Then, the ethanolic leaves extract of* D. multiflora* was collected in Eppendorf tube and preserved in a refrigerator at 4°C for further use.

### 2.3. Preparation of Working Solutions of Extracts

One hundred mg of* D. multiflora* extract was dissolved in 1 mL of sterile distilled water to a final concentration of 100 mg/mL. Thereafter, the solutions of* D. multiflora* extract used for the treatment were prepared at the doses of 111.42, 222.84, and 445.68 mg/kg corresponding to 8, 16, and 32 MIC, respectively [13]. The selected doses in this study were informed by the averages of daily consumed regimens recommended by traditional healers (no reported pharmacological study available). The solutions were homogenized using shaker and conserved in a freezer. Standard drug (ciprofloxacin) was also prepared at the dose of 2.5 mg/kg. The volume of solution to be administered was calculated using the following formula:

(1) $$\begin{array}{c} V=\frac{D\times W}{C}, \end{array}$$

where *V* is volume of extracted solution to be administered (mL), *D* is dose (mg/kg), *W* is animal weight (kg), and *C* is concentration of extract solution to be administered (mg/mL).

### 2.4. Microbial Strain

*Shigella flexneri *is commonly associated with diarrheal infection that actually presents a great public health burden because it is endemic and is the major causative agent of dysentery in developing countries [5]. This clinical isolate used to induce diarrhea in rats was obtained from “Centre Pasteur” in Yaounde, Cameroon. The bacterial strain kept at +4°C was activated before any test.

### 2.5. Preparation of Bacterial Inoculum

Direct colony suspension method was used in preparing the inoculum. Three to five morphologically similar colonies from fresh Muller Hinton Agar plates were transferred with a loop into about 5 mL of normal saline in a capped test tube and vortex. The suspension formed was adjusted to give a turbidity equivalent to that of a 4 McFarland standard (BaSO4 prepared spectrophotometrically) to give an approximate 1.2 × 10^9^ CFU/mL.

### 2.6. Experimental Animals

Male and female albino rats of Wistar breed (45 days old, weighing approximately 73 ± 19 g) not genetically modified were purchased from the Laboratory of Food Science and Metabolism and bred at the animal house of the Laboratory of Pharmacology and Toxicology, Department of Biochemistry, University of Yaoundé I, at room temperature for a 12 hours' light/dark photoperiod cycle. A 7-day acclimation period was observed before experiment. They were kept in their cages where they received standard diet and water ad libitum. The litter used was sawdust, renewed twice per week to ensure good hygienic status of animals.

### 2.7. Antidiarrheal Activity

This test was carried out on* S. flexneri*-induced diarrhea rats. Prior to the test, rats were fasted for 18 hours. All procedures performed were reviewed and approved by the Laboratory of Pharmacology and Toxicology, Department of Biochemistry, University of Yaounde I, and conform to the rules and regulations of the European Union on Animal care (CEE Council 86/609) [20] that were adopted by the institutional committee of the Ministry of Scientific Research and Innovation of Cameroon.

### 2.8. Diarrhea Induction

Diarrhea was induced by a suspension of* S. flexneri* prepared at 4 McFarland turbidity scale from an overnight* S. flexneri* culture on nutrient agar. 1 mL of this solution containing about 1.2 × 10^9^ cells/mL was orally administered to each animal [21]. Only infected animals were selected for the study.

### 2.9. Grouping of Animals

Thirty-six Wistar albino rats used for this study were divided into six groups of six animals each in separate cages. Apart from group 0 which had uninfected rats, the rest of the groups only had rats selected from the infected stock. Treatment was performed by administering the extracts orally, every morning for five days. The animals were treated as follows:

Group 0 was not infected and not treated and served as neutral control.

Group I was infected and not treated and served as negative control.

Group II received ciprofloxacin (2.5 mg/kg) and served as positive control.

Groups III, IV, and V received the* D. multiflora* extract at concentrations of 111.42 mg/kg, 222.84, and 445.68 mg/kg.

Standard diet and water were given to the animals before and during the treatment. Each day, the stools were collected just before administration of extract solution or ciprofloxacin to the test group of rats or water for the negative and neutral controls.

### 2.10. Assessment of Stool Bacterial Density

The extent to which the animals responded to the treatment was studied by counting the number of bacterial colonies forming units (CFU) in the stool samples. A sample of the stool (0.1 g) was completely dissolved in 5 mL of sterile distilled water. 50 *μ*L of the resulting solution was spread on the surface of solidified MacConkey Petri dishes. After incubation for 24 hours at 37°C, the number of colonies following growth of* S. flexneri* in each Petri dish was counted and recorded. The results were converted into the number of colonies per gram of stool per animal. The efficiency of treatment was assessed from the number of colonies obtained for each animal versus duration of treatment. This gave an idea on how the animals responded to the treatment using the extract and thus the duration of treatment using the optimum dose regiments.

### 2.11. Subacute Toxicity

The subacute toxicity study was carried out on the rats according to the Organization for Economic Cooperation and Development (OECD) guidelines on subacute toxicity [22] with slight modifications. Thirty male and female rats were weighed, marked orderly, and divided into three groups of 10 rats (5 of either sex) each. The plant extract was administered orally and daily for 14 days in single doses of 222.84 and 445.68 mg/kg corresponding to the therapeutic doses which showed the best antidiarrheal activity. The animals were treated as follows:

Group 0 was considered as the control group and only received distilled water.

Groups I and II received the extract solution of* D. multiflora* orally at the doses of 222.84 and 445.68 mg/kg, respectively.

### 2.12. Biochemical Parameters

At the end of the experiment, all rats were fasted overnight and sacrificed. Blood samples were collected in nonheparinized tubes and centrifuged at 3000 rpm to obtain the serum that served for the assessment of liver and kidney function parameters. The experiment was performed in accordance with protocols provided with commercial kits Fortress Diagnostics reviewed in October 2007. The levels of aspartate amino transferase (AST), alanine amino transferase (ALT), creatinine, glutathione, and total proteins were assessed.

### 2.13. Histopathological Study

All animals in this study were subjected to general autopsy. Animals were pinned down in a dissection tray by placing them with ventral side up. The abdominal skin was left with forceps and cut through with scissors. The scissor blade was close and inserted under the skin and moved in the cephalic direction. The rats were cut along the body midline, from the public region to the lower jaw. A lateral cut was made about halfway down the ventral surface of each limb. Liver and kidney were then cut, cleaned, and kept in the fridge. The relative weight of the liver and kidney was determined by the formula

(2) $$\begin{array}{c} Relative\;\;organ\;\;weight\left(\;\%\;\right)=\frac{weight\;\;of\;\;organ}{Animal\;\;weight}\times 100. \end{array}$$

The liver and kidney fixed in formaldehyde 10% for 3 weeks were cut into small pieces of 5 to 10 mm and then dehydrated in cassettes by immersion in alcohol and acetone successively. The latter was eliminated by xylol before being flowed in molds containing paraffin melted by heating at 60°C [23]. After cooling the strong block of paraffin containing cloth, it was cut with a microtome to achieve cuts of 5 *μ*m of thickness. The cuts were spread out glued-dried on blades in a steam during one night. They were colored by a solution of Haematoxylin-Eosin. After coloration the installation was made with Eukitt and the sample was placed between slide and slide-covers. The preparation was air-dried and the architectural aspects of kidney and liver were observed.

### 2.14. Statistical Analysis of Results

Data are expressed as mean ± standard error of the mean (SEM). The data were analysed using SPSS version 20. Statistical significance was determined by one-way analysis of variance (ANOVA) followed by post hoc Tukey's multiple comparison test. The value of *p* < 0.05 was considered statistically significant.

## 3. Results and Discussion

### 3.1. Antidiarrheal Activity

#### 3.1.1. General Signs of Diarrhea

Diarrheal stools appeared within 24 h after induction. The frequency and volume of diarrheal feces increased gradually in dose-dependent manner from day-1 to day-5 after induction and it appears soft with mucus or liquid or were molded and smooth. Sometimes, the presence of blood and mucus made the stool appear dark and shiny. The animals were weak, not as mobile, and curled up. Their coats were bristling and they were less aggressive, ate hardly, and were overcome with fatigue. These results confirm the effective infection of the rats [22, 24, 25]. Furthermore, the increase of the volume and the frequency of diarrheal stool observed are typical signs of intestinal invasion by* S. flexneri* [26]. This intestinal invasion by* S. flexneri *induces the production of an important oxygenated reactive metabolite, mediator of nitric oxide, which is accessory to the inflammation associated with diarrhea leading to general fatigue observed [27]. One rat from group I treated with the dose of 111.42 mg/kg of* D. multiflora *died after 2 days of treatment. Therefore only five animals (5/6) of this group were included in the analysis. No death was observed in groups O, II, III, IV, and V. Hence, six animals (6/6) of each group were included in the analysis. The importance of the metabolic activity of bacteria in the intestinal tract and toxins could be responsible for the animal death observed in group I [22].

#### 3.1.2. Effects of Diarrhea on the Body Weight

**Figure 1:**
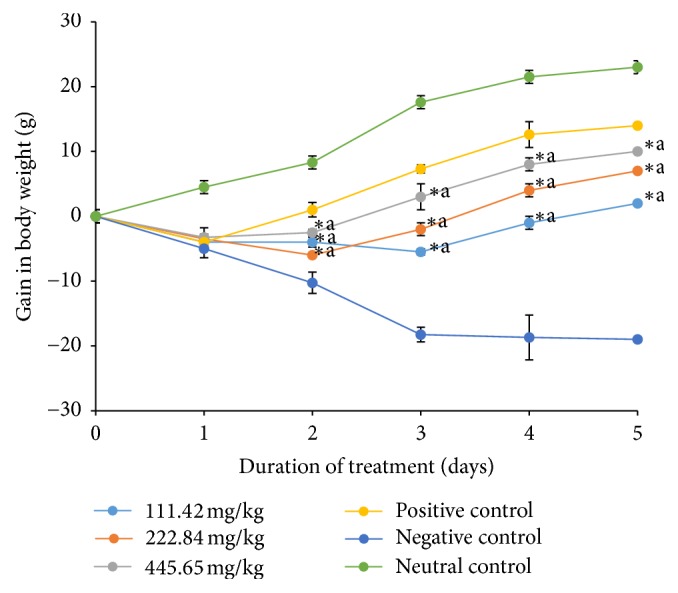
Weight gain of treated and untreated rats during treatment.* Shigella flexneri* diarrheic rats were treated for 5 days with 111.42 mg/kg, 222.84 mg/kg, and 445.68 mg/kg of ethanolic extract of* D. multiflora* or ciprofloxacin. Data are the mean ± SEM (*n* = 6). Significant difference: ^*∗*^*p* < 0.05 compared with negative control rats; ^a^*p* < 0.05 compared with positive control; day 0:* S. flexneri *administration; day 1: diarrhea appearance and treatment start.

The development of diarrhea was associated with nonsignificant (*p* < 0.05) weight loss which was observed for all infected groups on the second day of treatment. The resumption of weight gain was dose-dependent and significant (*p* < 0.05) at the doses of 222.48 and 445.68 mg/kg on the 2nd and 3rd day, respectively. The resumption of weight gain was observed on the 4th day of treatment in group treated with the dose of 111.42 mg/kg while no resumption of weight gain was noted with the negative control. No weight loss was noted in the neutral control group (Figure 1). Nevertheless, the weight gain in all the respective extract doses treated groups was found significantly (*p* < 0.05) lower when compared with the ciprofloxacin treated group. The weight loss associated with diarrheal installment could be due to the fact that this bacterium may have penetrated in the epithelial cells of the mucus membrane where it quickly multiplied and provoked the formation of abscesses and ulcerations, leading to the modification of intestinal reabsorption mechanisms [28].

#### 3.1.3. Effects of the Crude Extract on the Stool Load of* S. flexneri*

**Figure 2:**
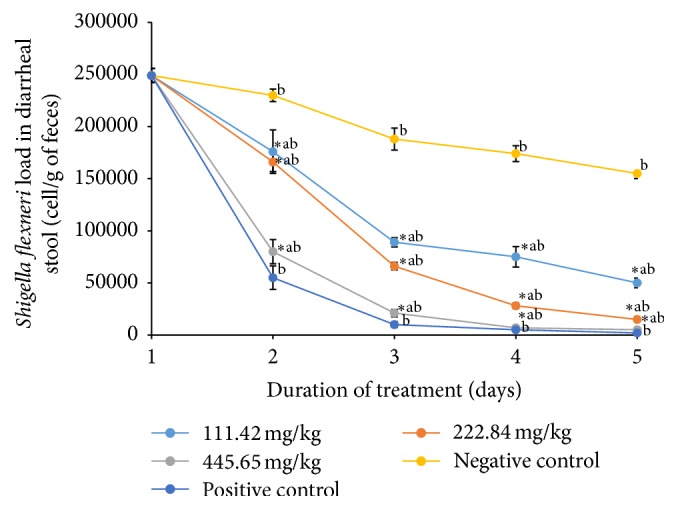
Variation of* S. flexneri* load in stools of treated and untreated rats during treatment. Rats were treated for 5 days with 111.42 mg/kg, 222.84 mg/kg, and 445.68 mg/kg of ethanolic extract of* D. multiflora* or ciprofloxacin. Data are the mean ± SEM (*n* = 6). Significant difference: ^*∗*^*p* < 0.05 compared with negative control; ^a^*p* < 0.05 compared with positive control; ^b^*p* < 0.05 compared with initial point; day 1: diarrhea appearance and treatment start.

The treatment improved the general condition of the rats. Treatment of infected rats with the plant extract and ciprofloxacin significantly decreased (*p* < 0.05)* S. flexneri* stool load compared to the negative control. The evidence of antidiarrheal activity was noted by the decrease in bacterial load in the diarrheal stool. The number of* S. flexneri* CFU/gram of stool as a function of duration of treatment dropped significantly in a dose-dependent manner during treatment (Figure 2). Animals stopped manifesting diarrhea after three and four days of treatment with the doses of 445.68 mg/kg and 222.84 mg/kg of ethanolic extract of* D. multiflora*, respectively, and persisted even after day-5 with the minimum dose of 111.42 mg/kg. The ciprofloxacin stopped the diarrhea after 2 days of treatment. In the case of the negative control, the number of colonies counted per gram of stool remained very high after 5 days of treatment. However, it was observed that, from day-1, the number of colonies started declining gradually. Groups treated with the plant extract recorded significantly higher (*p* < 0.05) colony counts than group of rats treated with ciprofloxacin, while rats treated with 445.68 mg/kg of the plant extract had total clearance by the 5th day of treatment. The higher activity of ciprofloxacin compared to that of the extract could be explained based on the fact that ciprofloxacin is a pure compound which may act directly on the target site while crude extract contains a mixture of compounds which together may not allow the active compounds to be more efficient. Tannins, alkaloids, saponins, steroids, and terpenoids identified in the ethanolic crude extract were found to be responsible for the* in vitro* antibacterial activity observed on* S. flexneri* [13]. It was shown that tannins and tannic acid present in antidiarrheal plants denature proteins in the intestinal mucosa by forming precipitate which makes the intestinal mucosa more resistant to chemical alteration and reduces secretion [29]. The antisecretion potentials of tannins could contribute to the observation of antidiarrheal activity of* D. multiflora*. These phytochemicals present in extract could therefore justify its antidiarrheal activity. The antidiarrheal activity may be associated with the antimicrobial activity of these extracts [30]. Furthermore, ethanolic leaves extract of* D. multiflora* at the doses of 445.68 and 222.84 mg/kg significantly decreased the frequency of defecation and wetness of the feces suggesting an activity similar to that of ciprofloxacin. It was also observed that the stool load of* S. flexneri* decreased in the nontreated group; this could be attributed to the installation of an immune response against the foreign microbe [31]. To the best of our knowledge, no previous study has reported the antidiarrheal activity of ethanolic extract of* D. multiflora*. However antidiarrheal activity of aqueous and methanolic leaves extracts of* Dissotis thollonii* Cogn. (Melastomataceae), a plant of the same family, showed a reduction in the bacterial load from day-2 of treatment in all infected animal stool treated at different doses of the extracts (500, 250, and 125 mg/kg) [16].

### 3.2. Subacute Toxicity Test

#### 3.2.1. General Signs of Subacute Toxicity

Toxicological evaluations after repeated exposures are required by regulatory agencies to characterize the toxicological status of any substance [22]. However, there is no toxicological information in the literature to support and ensure the safe use of* D. multiflora* leaves extract. The present study represents the first research that demonstrates the subacute toxicity of* D. multiflora* leaves extract in male and female rats. The 14 days' daily repeated dose treatment with the doses of 222.84 and 445.68 mg/kg of the ethanolic extract of* D. multiflora* led to neither death nor visual signs of toxicity in any of the test groups throughout the treatment period. It has been shown that daily dose treatment with the extract which elicited no clinical signs of toxicity, morbidity, or mortality across all the treatment groups may be a tenable inference that the extract is unlikely to be toxic at the tested doses over the observation period [32]. However, it is not possible to generalize that the species* D. multiflora* has no toxicity based on this observation.

#### 3.2.2. Effects of the Crude Extract on the Body Weight

The respective initial body weights of the treated rats and the control were compared with their final weights. A relative increase of body weight was noted in all treated and untreated groups during this study. However, male and female rats treated with the doses of 222.84 and 445.68 mg/kg of the ethanolic extract of* D. multiflora* had lower weight gain (*p* < 0.05) when compared to those of the control groups (Table 1). Toxicity study at repeated doses for 14 days revealed that the dose of extract used for the treatment may exert an effect on some parameters in rats. Rats treated with various doses of ethanolic extract of* D. multiflora* had a progressive weight gained. This progressive increase in body weight at doses of 222.84 and 445.68 mg/kg of female and male rats during 14 days of administration of ethanolic extract of* D. multiflora* may indicate the improvement of the nutritional state of the animal during treatment. The growth response effect could be as a result of increased food and water intake. Nevertheless, the weight gain is significantly (*p* < 0.05) lower from that of the control in dose-dependent manner. This observation could suggest probable dose/absorption interactions [33] and possible anorexic properties of the ethanolic extract of* D. multiflora*.

#### 3.2.3. Effects of Ethanolic Extract of* D. multiflora* on Some Biochemical Parameters

The liver and kidney are two important organs which play key roles in the metabolic pathways of the body. The liver is the site of drug metabolism while the kidney ensures drug reabsorption and excretion [34]. ALT and AST are two transaminases found at a high level in the liver. They are used as biomarkers for predicting possible toxicity [35]. In male rats treated with the dose of 445.68 mg/kg of* D. multiflora*, a significant increase (*p* < 0.05) of the mean level of ALT and creatinine was noted with respect to the control while a significant increase of the level of creatinine (*p* < 0.05) was noted in rats treated with the dose of 222.84 mg/kg. In female rats, a significant decrease (*p* < 0.05) of the mean level of AST and creatinine was noted in rats group treated with the dose of 222.84 mg/kg of* D. multiflora* with respect to the control. However, no significant increase (*p* < 0.05) was noted in the level of glutathione (Table 2). It suggests that this extract did not generate oxidative stress in rats. The increase of the level of ALT and AST underlines a hepatic cell lysis which is an indication of a likelihood of the toxicity of this organ due to the treatment of rats with the extracts [36]. Creatinine is an important marker for kidney dysfunction and the increase in serum creatinine levels is associated with kidney dysfunction [37]. This finding suggests that the ethanolic extract of* D. multiflora* is toxic at the doses of 222.84 and 445.68 mg/kg. Therefore, further investigation is needed to verify this finding.

#### 3.2.4. Effects of the Crude Extract on the Relative Weight (%) of Organs

Relative organ weight is an important index to determine whether the organ has been exposed to injury [38]. No significant difference (*p* < 0.05) in the weight of organs (liver and kidney) of treated rats was noted at the doses of 222.84 and 445.68 mg/kg compared to control group (Table 3). There were no significant (*p* < 0.05) variations in relative organ weights between groups. Based on this result, no organ toxicity occurred since previous works have showed that an increase in rats' organs after exposure to plant extracts is a sign of toxicity [39]. However, the observed nonsignificant (*p* < 0.05) variations in relative organ weights between groups are not sufficient to support that the ethanolic leaves extracts of* D. multiflora* have no toxicity.

#### 3.2.5. Histological Exams

**Figure 3:**
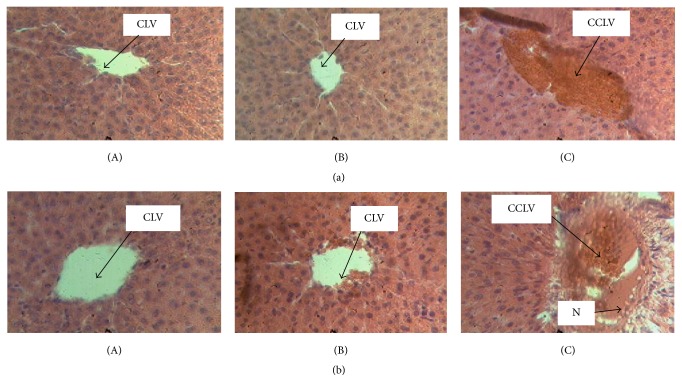
Histological section of liver tissue (section stained with H&E, ×400). (A) Control with normal hepatocytes and centrilobular vein. (B) Liver of rats treated at the dose of 222.84 mg/kg of* D. multiflora* without capillary sinusoids dilations in both sexes and slight capillary sinusoids dilations in male treated rats. (C) Liver of rats treated at the dose of 445.68 mg/kg of* D. multiflora* with vascular congestion + slight capillary sinusoids dilations. (a) = male; (b) = female; CCLV = congestion of centrilobular vein; CLV = centrilobular vein; N = necrosis; H&E: Haematoxylin-Eosin.

**Figure 4:**
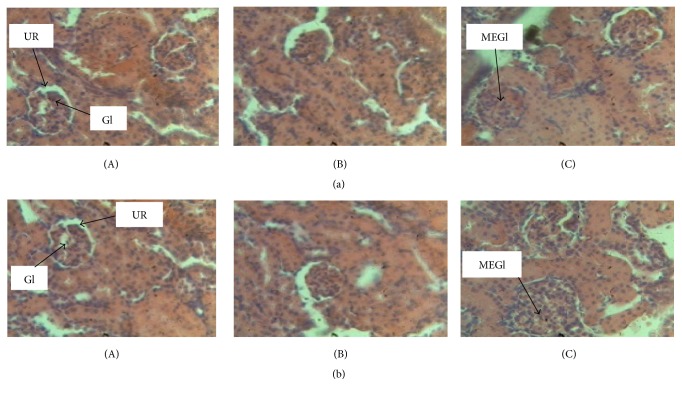
Histological section of kidney tissue (section stained with H&E, ×400). (A) Control with normal urinary room and glomerulus. (B) Kidney of rats treated at the dose of 222.84 mg/kg of* D. multiflora* with normal aspect without mesangial expansion. (C) Kidney of rats treated at the dose of 445.68 mg/kg of* D. multiflora* with mesangial expansion and urinary room presenting some alterations. (a) = male; (b) = female; MEGl = mesangial expansion of glomerulus; UR = urinary room; Gl = glomerulus; H&E: Haematoxylin-Eosin.

The histological examination of the livers and kidneys showed that the livers of male and female rats of the control group were normal with hepatocytes separated by narrow sinusoids. Livers of male and female rats treated at the dose of 222.84 mg/kg of* D. multiflora* appear normal without capillary sinusoids dilations in both sexes. Rats treated at the dose of 445.68 mg/kg of ethanolic leaves extract of* D. multiflora* presented degenerative modifications of livers: the architecture of the hepatic tissue was found to be partially erased, sinusoids were dilated, and veins marked the vascular congestions of the liver (Figure 3). The histological examination of the kidney of male and female rats of the control group and those of the group treated at the dose of 222.84 mg/kg of* D. multiflora* showed no alterations of urinary room and glomerulus while rats treated at the dose of 445.68 mg/kg of* D. multiflora* presented mesangial expansion of glomerulus and some alterations of urinary room (Figure 4). Previous works showed that such hepatic and renal changes are misled by other plants containing some poisonous molecules [40]. However, results indicate a starting necrosis that can in the long-run lead to a total hepatic cell necrosis. It is evident from these observations that ethanolic crude extract of* D. multiflora* induces liver and kidney damage at the dose of 445.68 mg/kg while no major damage is observed at the dose of 222.84 mg/ml. This result suggests that the ethanolic extract of* D. multiflora* is not toxic at the dose of 222.84 mg/ml. However, since this study revealed an increase in creatinine level in both male and female rats, one cannot assert that this extract is nontoxic at the dose of 222.84 mg/kg. It is therefore evident that this extract is much toxic at the dose of 445.68 mg/kg. Toxicological evaluation of the ethanolic extract of* D. multiflora* is reported herein for the first time. That notwithstanding previous work on subacute toxicity of ethanolic extract of a plant of the same genera, namely,* Dissotis rotundifolia (Melastomataceae)*, showed an obvious toxicity after oral administration of repeated dose of 500 mg/kg to rats and that the treatment using a lower dose is therefore advisable [18]. Our finding did not corroborate their hypothesis since the results obtained in this study showed that the ethanolic extract of* D. multiflora* alters the liver and renal function and further supports the toxic nature of* D. multiflora* extract at the dose of 222.84 and 445.68 mg/kg and is most pronounced at the dose of 445.68 mg/kg.

## 4. Conclusion

This finding justifies the use of* D. multiflora* leaves extract in the traditional treatment of diarrhea. The efficient treatment doses for infectious diarrhea caused by* S. flexneri* are 222.84 and 445.68 mg/kg. However, this extract is toxic at both doses and most pronounced at the dose of 445.68 mg/kg. Therefore the treatment with doses lower than 222.84 mg/kg is recommended while further study is required to define the exact efficient nontoxic dose.

## Figures and Tables

**Table 1:** Variation of body weight gain (g) for treated and untreated ratsduring treatment.

Sex	Doses (mg/kg)	Body weight (g)			% weight gain
		Day 0	Day 7	Day 14	
Male	0	65.5 ± 2.12	80 ± 0.48	92.6 ± 1.50	41.37
	222.84	58.8 ± 0.10	71.6 ± 2.52	76.1 ± 1.73	29.42^*∗*^
	445.68	55.8 ± 1.68	65 ± 1.11	70 ± 0.75	25.44^*∗*^

Female	0	68.4 ± 2.76	79.6 ± 2.05	88.4 ± 2.89	29.23
	222.84	70 ± 1.45	79.2 ± 1.82	83.2 ± 0.48	18.85^*∗*^
	445.68	64 ± 1.56	70.8 ± 1.75	75.5 ± 0.9	17.96^*∗*^

The results are mean ± standard error of mean (SEM) (*n* = 5). Data in the same column in the same sex with different superscript are significantly different (*p* < 0.05) when compared to the control.

**Table 2:** Effects of ethanolic extract of *D. multiflora* on some biochemical parameters.

Sex	Doses	ALT	AST	TP	GSH	CREA
	(mg/kg)	(U/L)	(U/L)	(g/L)	(g/L)	(g/L)
Female	00	9.21 ± 0.68	19.34 ± 0.69	38.78 ± 0.39	0.02 ± 0.00	0.46 ± 0.03
	222.84	8.36 ± 0.35	13.44 ± 1.61^*∗*^	38.90 ± 0.25	0.02 ± 0.00	0.39 ± 0.02^*∗*^
	445.68	9.07 ± 0.2	22.35 ± 1.1	41.83 ± 1.72	0.02 ± 0.00	0.49 ± 0.01

Male	00	8.43 ± 0.21	15.13 ± 1.08	40.29 ± 1.44	0.03 ± 0.00	0.33 ± 0.01
	222.84	7.53 ± 0.27	12.99 ± 0.90	38.22 ± 0.99	0.02 ± 0.00	0.48 ± 0.02^*∗*^
	445.68	10.86 ± 0.24^*∗*^	17.39 ± 0.66	39.85 ± 1.52	0.02 ± 0.00	0.52 ± 0.03^*∗*^

The results are mean ± standard error of mean (SEM) (*n* = 5). Data in the same column in the same sex with different superscript are significantly different (*p* < 0.05) for all treated groups compared with the control group. ALT: alanine amino transferase (U/L); AST: aspartate amino transferase (U/L); TP: total proteins (g/L); GSH: glutathione (g/L); CREA: creatinine (g/L).

**Table 3:** Relative organs' weight (%) of treated and untreated rats.

Sex	Doses (mg/kg)	Relative organ weights (% body weight)	
		Liver (g)	Kidney (g)
Female	0	3.20 ± 0.33	0.78 ± 0.00
	222.84	3.20 ± 0.00	0.80 ± 0.03
	445.68	3.40 ± 0.33	0.86 ± 0.06

Male	0	3.33 ± 0.33	0.80 ± 0.00
	222.84	3.40 ± 0.33	0.83 ± 0.03
	445.68	3.60 ± 0.33	0.86 ± 0.06

The results are mean ± standard error of mean (SEM) (*n* = 5). Data in the same column in the same sex were considered significantly different for *p* < 0.05 for all treated groups compared with the control group.
